# Case series: Montgomery T-tube placement for subglottic tracheal stenosis: a report of 3 cases

**DOI:** 10.1097/MD.0000000000032680

**Published:** 2023-01-13

**Authors:** Ping-Yang Hong, Mao-Hong Huang, Feng-Fu Zhan, Yi-Li Lin, Shao-Zhao Qiu, Xiao-Bin Zhang

**Affiliations:** a Department of Pulmonary and Critical Care Medicine, Zhongshan Hospital of Xiamen University, School of Medicine, Xiamen University; The Third Clinical College of Fujian Medical University; Key Clinical Specialty of Fujian Province, Fujian, China; b Department of Anesthesia, Zhongshan Hospital of Xiamen University, School of Medicine, Xiamen University; The Third Clinical College of Fujian Medical University, Fujian, China.

**Keywords:** montgomery T-tube, subglottic, tracheal stenosis, tracheostomy tube

## Abstract

Subglottic tracheal stenosis is stenosis of the trachea between the vocal cords and the lower margin of the cricoid cartilage. The Montgomery T-tube is used as a tracheostomy tube and a combined tracheal stent to avoid postoperative tracheal stenosis.

**Patient concerns:**

Because the stenosis is close to the glottis, surgical treatment is complex, and many complications may arise.

**Diagnoses:**

Subglottic tracheal stenosis.

**Interventions:**

The patients underwent endotracheal intubation or tracheotomy because of acute pancreatitis, laryngeal malignancy, or cerebral hemorrhage after endotracheal intubation or tracheotomy and presented with varying degrees of tracheal stenosis and dyspnea. We relieved airway stenosis and improved dyspnea in these 3 patients by placing a Montgomery T-tube.

**Outcomes:**

None of the 3 patients had intraoperative complications. In 2 of the cases, airway secretions were stored after surgery.

**Lessons:**

Montgomery T-tube placement is safe and effective for patients with complex subglottic tracheal stenosis.

## 1. Introduction

Subglottic tracheal stenosis refers to stenosis of the trachea between the lower edge of the vocal cord and the lower edge of the cricoid cartilage (within 20 mm of the glottis).^[1,2]^ Because the stenosis is close to the glottis, surgical treatment is complex, and many complications may arise.^[3]^ Endoscopic interventional treatments, such as electrosurgical release, balloon dilatation, and freezing, effectively treat membranous subglottic tracheal stenosis.^[4]^ However, complex subglottic stenosis (stenosis length ≥10 mm with softening collapse or tracheal atresia) often requires stent placement. Silicone stent placement can cause serious complications, such as granulation hyperplasia and displacement, which is a challenging problem in clinical practice.^[5]^ Recently, placing a Montgomery T-tube during respiratory intervention has become an important treatment option for such patients.^[6,7]^ This article reviews and reports the clinical data of patients with complex subglottic tracheal stenosis and T-tube placement in our hospital.

## 2. Case presentation

The characteristics of the 3 patients are shown in Table 1.

**Table 1:** Patients’ characteristics.

	Case 1	Case 2	Case 3
Age (yr)	20	65	72
Sex	Male	Male	Female
Cause	Pancreatitis	Laryngeal cancer	Cerebral hemorrhage
Complications	Diabetes and hyperuricemia	Diabetes, chronic obstructive pulmonary disease, and hypertension	Diabetes and hypertension
Duration of endotracheal tube (d)	13	0	10
Duration of tracheotomy (d)	18	9	371
The length of the stenosis (cm)	2.2	3.1	2.5
Diameter of the trachea at its narrowest point (cm)	0.3	0.5	0.4
Prior airway surgeries	Electrocoagulation, laser, cryoablation, silicone stent, and balloon dilation	Balloon dilation	Laser and balloon dilation
Anesthesia method	General anesthesia	General anesthesia	General anesthesia
Selection of tracheoscope	Rigid bronchoscope in size 14	Rigid bronchoscope in size 14	Rigid bronchoscope in size 12
The main manifestations of tracheoscopy	Granulation tissue	Left vocal cord fixation	Granulation tissue
The stenosis was improved	Yes	Yes	Yes
Able to pronounce	Yes	Yes	No
Attempts at decannulation	No	No	No
Intraoperative complications	No	No	No
Postoperative complications	No	Retention of airway secretions	Retention of airway secretions

### 2.1. Case 1

**Figure 1.**
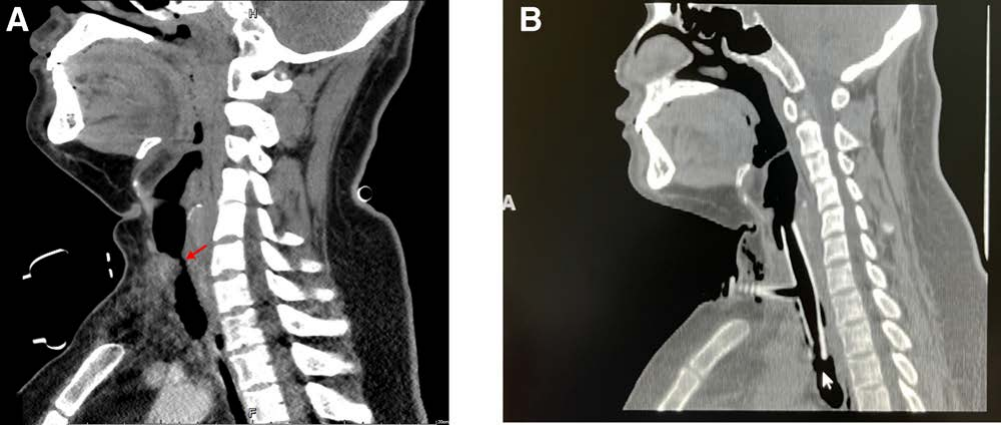
Before and after Montgomery T-tube placement in case 1. (A) The subglottic tracheal lumen was significantly deformed and narrowed. The narrowest diameter was ~0.3 cm and the upper and lower range was approximately 2.2 cm (before treatment). (B) After Montgomery T-tube placement.

**Figure 2.**
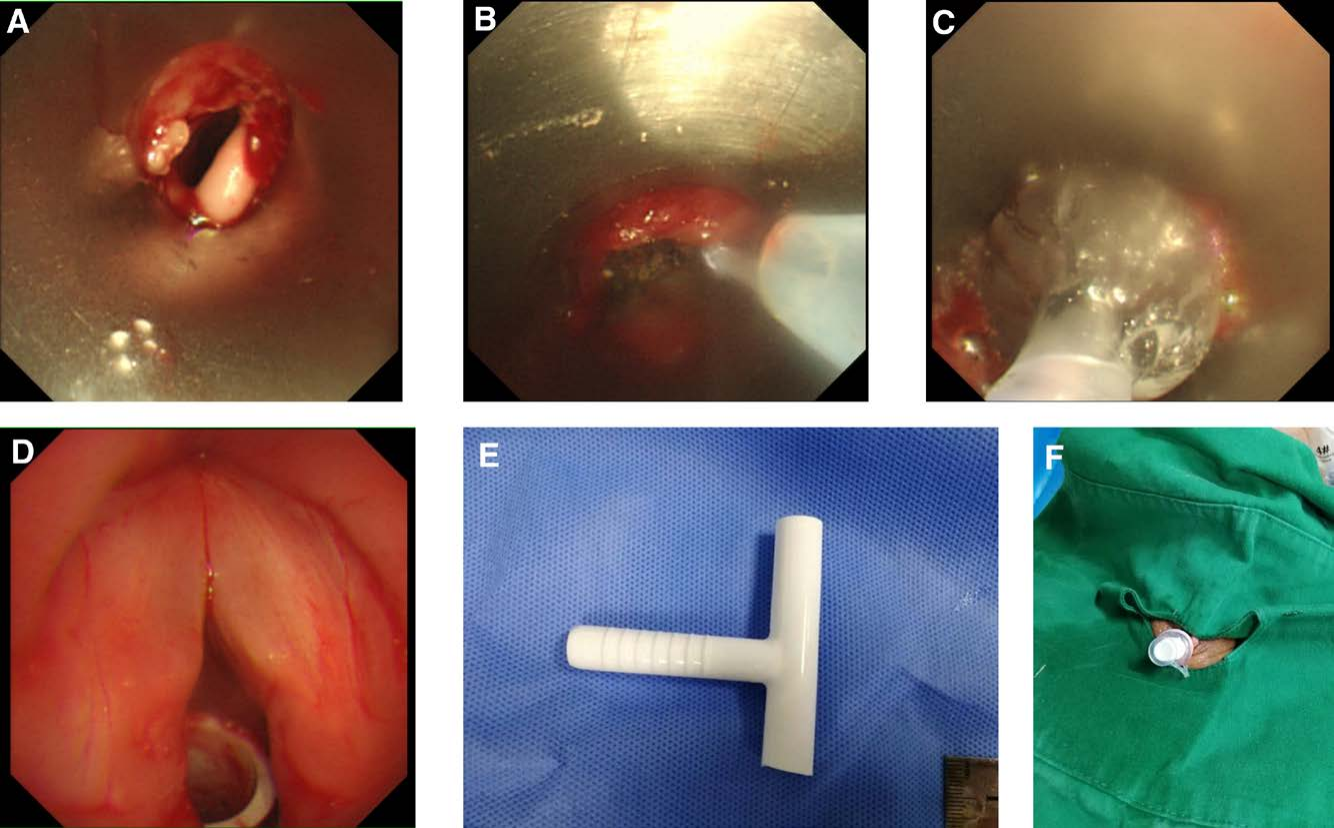
Montgomery T-tube placement procedure in case 1. (A) Subglottic stenosis. (B) Laser ablation. (C) Balloon dilatation. (D) Above the Montgomery T-tube. (E) Montgomery T-tube. (F) After Montgomery T-tube placement.

A 20-year-old man was admitted to our hospital for acute pancreatitis and underwent endotracheal intubation. Three months after endotracheal intubation, an emergency CT examination of the neck revealed severe subglottic stenosis, classified as Cotton Myer grade IV^[8]^ (Fig. 1). The patient’s shortness of breath was significant, and the blood gas analysis results suggested respiratory failure; therefore, tracheotomy was performed in the emergency intensive care unit. After evaluation, the patient underwent laser ablation for airway scarring and balloon dilatation of the narrowed airway. Transbronchoscopic laser ablation, cryoablation, balloon dilatation, and silicone stent implantation were performed under general anesthesia. Two weeks after the tracheotomy, the patient underwent Montgomery T-tube placement. The 14 mm silicone Montgomery T-tube was cut to 4 cm at the top and 4.5 cm at the bottom. The upper branch of the T-tube was approximately 1.5 cm from the glottis. The placement proceeded smoothly (Fig. 1). Montgomery T-tube placement was treated as the transitional phase for this patient (Fig. 2). Patients are regularly reviewed at the hospital and atomized regularly. We plan to evaluate the patient 2 years after the procedure to determine whether the Montgomery T-tube could be removed.

### 2.2. Case 2

A 65-year-old man was diagnosed with laryngeal cancer and underwent surgery. Nine months postoperatively, the patient experienced progressive dyspnea. Stereoscopic examination revealed a stenosis of the glottic fissure. Emergency tracheotomy under local anesthesia was performed, followed by anti-infection and atomization treatment. The patient’s dyspnea improved significantly.

Nevertheless, the patient was uncomfortable with the tracheotomy tube and wanted to pronounce it. Nine days after the emergency tracheotomy, the patient underwent Montgomery T-tube placement. A Montgomery T-tube was inserted under general anesthesia as it is more conducive to surgery. A high-frequency jet ventilator was connected after insertion of a rigid bronchoscope. An umbilical string was inserted through the external branch of the Montgomery T-tube and pulled out from the proximal end, and the string at the proximal end of the Montgomery T-tube was pulled out through the tracheostomy stoma in the mouth through a rigid bronchoscope. The patient felt comfortable with the Montgomery T-tube insertion and was able to pronounce it freely.

### 2.3. Case 3

A 72-year-old woman underwent tracheotomy for pulmonary infection after intracerebral hematoma removal and decompressive craniectomy. One year after the tracheotomy, the patient’s family requested removal of the tracheostomy cannula after long-term indwelling of the tracheostomy catheter. A year and 1 month after the tracheotomy, electronic bronchoscopy revealed granulation tissue formation at the upper and lower edges of the tracheotomy cannula. However, our attempt to remove the tracheal cannula failed. Six days after the initial bronchoscopy, the patient underwent bronchoscopic laser ablation of the granulation tissue and Montgomery T-tube implantation. The surgery proceeded smoothly, and the patient recovered well after discharge. The patient was able to close the Montgomery T-tube collaterals while breathing through the nasopharynx, and no neck tissue infection occurred.

## 3. Discussion and conclusions

Tracheal stenosis confined to the subglottic area is mainly caused by endotracheal intubation or incision.^[4]^ Tracheal stenosis in our 3 cases here was due to tracheal intubation/incision. Transmural ischemic injury to the trachea has been observed after endotracheal intubation/incision.^[1]^ Scar stenosis formed during injury repair is known as post-intubation tracheal stenosis.^[9]^ The risk factors for tracheal intubation complicated by tracheal stenosis include violent intubation, prolonged intubation time, and excessive pressure in the intubation sleeve.^[4]^ Tracheotomy complicated by tracheal stenosis is affected by multiple factors, including patient age, operative mode, duration of mechanical ventilation, complicated respiratory tract infection, diabetes mellitus, preoperative intubation time, and gastroesophageal reflux.^[10]^ In the third case, the tracheotomy was approximately 10 months, and the patient was elderly and complicated with diabetes. These are risk factors for tracheal stenosis after tracheostomy.

Treatment of subglottic tracheal stenosis includes surgical and endoscopic intervention.^[5]^ A combination of treatments can be used for different types and lengths of stenosis. Stenting is initiated when the efficacy of multiple interventional methods is not satisfactory, the trachea cannot maintain stable patency, the trachea is malacic or collapsed, and surgical treatment is not available or not ready.^[11]^ The Dumon silicone stent is preferred for benign tracheal stenosis,^[12]^ but the Montgomery T-tube is more advantageous than the Dumon silicone stent for subglottic benign tracheal stenosis.^[4]^ All patients in this study had complex subglottic stenosis. Patients 1 and 2 received endoscopic interventional treatment before Montgomery T-tube implantation, including ablation, freezing, mechanical dilation, and stent placement; however, the effect was poor, and surgery was refused.

The main advantages of the Montgomery T-tube over the tracheostomy tube are as follows^[6,13]^: the side branches of the Montgomery T-tube can be closed by breathing through the normal nasopharynx; a Montgomery T-tube smaller than the trachea can be used to reduce granulation; the patient was able to pronounce this; and sputum suction was more convenient. However, the Montgomery T-tube has some disadvantages^[4]^: it is unsuitable for patients requiring positive airway pressure ventilation; patients may be unable to discharge sputum smoothly; and the Montgomery T-tube is unsuitable for patients with recurrent lung infections. In case 1, the Montgomery T-tube was mainly used for interim treatment, and we eventually planned to remove the Montgomery T-tube and close the patient’s neck fistula. The patient in case 2 had a malignant tumor of the larynx. Because the patient had a strong need for vocalization, T-tube placement was performed. In case 3, because the tracheotomy time was >1 year old, there were many granulation tissues in the tracheotomy catheter. Replacement of the Montgomery T-tube significantly reduced granulation, allowing the patient to breathe through the mouth and nose.

The main complications after Montgomery T-tube implantation can be divided into surgery-related and long-term indwelling Montgomery T-tube complications. Because Montgomery T-tube placement in endoscopic interventional therapy is generally performed after the tracheostomy sinus is stable, surgery-related complications are significantly reduced compared to surgical treatment.^[4]^ Complications are mainly related to the complexity of the surgical procedure, most of which occur in patients with grade III to IV subglottic stenosis.^[7]^ Due to severe tracheal stenosis or occlusion, it is challenging to dredge the trachea before placing a Montgomery T-tube.^[3,7]^ The main complications are tear of the tracheal mucosa, complication with mediastinal emphysema, and pneumothorax. None of the 3 patients had postoperative complications.

Montgomery T-tube displacement was not observed in this group. The advantage of the Montgomery T-tube over other airway stents because it is immobilized during tracheostomy with slight displacement. In general, a Montgomery T-tube is safe and effective.

## Author contributions

**Formal analysis:** Yi-Li Lin.

**Funding acquisition:** Ping-Yang Hong, Xiao-Bin Zhang.

**Investigation:** Ping-Yang Hong.

**Methodology:** Mao-Hong Huang.

**Project administration:** Feng-Fu Zhan, Xiao-Bin Zhang.

**Resources:** Shao-Zhao Qiu.
